# Correlation of Materials Property and Performance with Internal Structures Evolvement Revealed by Laboratory X-ray Tomography

**DOI:** 10.3390/ma11101795

**Published:** 2018-09-21

**Authors:** Lei Zhang, Shaogang Wang

**Affiliations:** Shenyang National Laboratory for Materials Science, Institute of Metal Research, Chinese Academy of Sciences, Shenyang 110016, China

**Keywords:** X-ray tomography, in situ, mechanics, corrosion, biomaterial, battery

## Abstract

Although X-rays generated from a laboratory-based tube cannot be compared with synchrotron radiation in brilliance and monochromaticity, they are still viable and accessible in-house for ex situ or interrupted in situ X-ray tomography. This review mainly demonstrates recent works using laboratory X-ray tomography coupled with the measurements of properties or performance testing under various conditions, such as thermal, stress, or electric fields. Evolvements of correlated internal structures for some typical materials were uncovered. The damage features in a graded metallic 3D mesh and a metallic glass under mechanical loading were revealed and investigated. Micro-voids with thermal treatment and void healing phenomenon with electropulsing were clearly demonstrated and quantitatively analyzed. The substance transfer around an electrode of a Li-S battery and the protective performance of a Fe-based metallic glass coating on stainless steel were monitored through electrochemical processes. It was shown that in situ studies of the laboratory X-ray tomography were suitable for the investigation of structure change under controlled conditions and environments. An extension of the research for in situ laboratory X-ray tomography can be expected with supplementary novel techniques for internal strain, global 3D grain orientation, and a fast tomography strategy.

## 1. Introduction

It is of common interest to understand the properties or performance of materials with their underlying structures in the corresponding length scales from atomic to macro in materials research. In practice, the structures of materials are examined by sampling before or after the measurements of the properties or performance testing. Samples need to be cut and carefully prepared to fulfill various analytical instruments by which the structural information can be obtained. Then, the researcher explains or deduces the material properties from the data collected statically. Technical obstacles make it difficult to understand the properties of the materials or the performances involved in changes in internal structures during their measurement or testing in real conditions and living states. 

Images, patterns, and spectroscopy are commonly employed to show the material’s structure through morphology, crystallography, or chemistry. These techniques of microanalysis originate from the reflection, diffraction, and interaction of materials with physical probes, such as light, lasers, X-rays, electrons, neutrons, or ions, etc. By manipulating these probes on a micro and nano scale, microanalysis can provide two-dimensional (2D) data of the material’s structure with spatial resolution in terms of the interaction volume. Researchers are likely to use this individual cross-section of the material structure to relate to the property of the bulk material. This works reasonably well for properties related to homogenous or uniformly distributed structure, for example, the elastic modulus of structural materials. However, the structure of the sliced material may miss some critical information on local or heterogeneous structural variation on which the property of the material relies. Crack growth in composites and damage induced by void accumulation in plastic deformation are two obvious cases [1]. Tomography is a state-of-the-art technique to complement the ordinary characterization of the planar structure with the spatial structure in three dimensions (3D). With the increases in computer power and software resources, it is now feasible to reconstruct a 3D digital volume of a sample with an enormous amount of planar structural data. One can use either stacked tomography from sliced structural data produced layer by layer or computed tomography (CT) from projections with the rotation angles downstream to the probe beam transmission of the sample. 

The stacked tomography by optical microscopy (OM) and scanning electron microscopy (SEM) need a number of consecutive sections from a sample surface, called serial sections, which can be performed by microtomy, ion milling, or consecutive polishing steps [2]. When accompanied with a focused ion beam (FIB) or a plasma FIB [3], recently-developed SEM tomography can be made more automatic while the data collection is interlaced with removing of material layer-by-layer on the sample surface. Practically, the spatial resolution can be as high as 10 nm with FIB serial sectioning. So far atom probe tomography (APT) is the most advanced technique producing a 3D volume composed of atom species in stacked tomography [4]. In practice, the outmost atomic layer at the tip of a needle shaped sample can be ionized and kicked off layer by layer in a pulsed high-voltage field. A flat panel time-of-flight (TOF) detector continuously collects the atomic-layer signals in sequence, from which the tip of the sample can be reconstructed [5,6]. However, the preparation of the needle sample with tens of nanometers in diameter needs skillful thinning and sharpening techniques. These stacked tomography techniques are inevitably destructive. In computed tomography, transmission electron microscopy (TEM) combined with CT technique, 3D-TEM, can reconstruct in 3D the volume with a resolution down to the nanometer range [7,8]. However, the sample has to be thinned down to tens of nanometers for the penetration of the high-voltage accelerated electrons. X-ray tomography (XRT) is another popular class in computed tomography. The obvious feature of XRT is that it is nondestructive. The XRT technique is, nowadays, commonly employed for clinical diagnosis as X-rays can easily penetrate biological tissues and bones. It can also be used for materials analysis through optional higher energy X-rays suitable for the penetration of specified materials and sample thicknesses.

### 1.1. XRT and In Situ Experiment

The invention and development of XRT for materials research since the last century has been reviewed in detail [9,10]. XRT has been widely used in the characterization of internal structures for various materials. It covers traditional materials including polymers, ceramics, metals and alloys, and also advanced materials with complex structures, such as composites [11,12,13], foams and cellular structures [14,15,16], biomaterials [17,18,19], etc. The recent development of XRT techniques have been reviewed comprehensively [20] and reach specified characterizations not only for internal damage [1], but also grain orientation [21,22,23,24], chemical information [25], and even internal strain [26] with a global 3D view. 

In addition to the ability to show the internal structure of a wide variety of materials in static states, the capability of XRT to set test conditions facilitates two paralleled processes of both 3D imaging of internal structures and measurement of properties in dynamic states. Their relationship can be correlated more easily and directly. Pioneering works of the in situ XRT in materials science can be traced back to the 1990s. Guvenilir et al. investigated the crack evolution in an aluminum–lithium alloy employing XRT combined with in situ loading experiments [27]. Since then, the damage inside materials induced by processing, cycling, or deformation have been investigated from 3D viewpoint. Vivid 3D structures of materials and quantitative analysis of the temporal state provide valuable insights as material properties rely on microstructure evolvement. Research works are still very active and versatile in the experimental mechanics field. Deformation and fracture are still common interest for metals and alloys [28,29,30,31,32,33,34,35,36,37,38,39,40,41,42], foams and porous materials [11,14,43,44,45,46], and composites [13,47,48,49]. XRT has also been considerably extended with other in situ measurements including materials processing [50,51,52,53,54,55], materials interaction with specified conditions involving extreme temperature [56,57], corrosion [58,59,60], electrochemical environments, as in batteries [61,62,63], etc. The finite element (FE) method has also been employed to analyze the dynamic processes with the change of applied fields based on 3D models from XRT [16,64,65,66]. Detailed reviews have also been published on in situ XRT for materials science [20,67], which clearly shows the fast growth of the technique in the past two decades. Such in situ experiments usually provide comprehensive 3D quantitative data and related properties or the performance of materials.

### 1.2. Laboratory-Based and Synchrotron Radiation XRT

X-rays from either a laboratory-based (LB) tube or synchrotron radiation (SR) are available to carry out XRT with in situ experiments. Due to the X-ray characteristics from different sources, the instrumentation for a specified experiment has to be considered beforehand. 

On one hand, X-ray source and imaging methods affect the quality of XRT for fine structures. LB-XRT usually uses radiography or cone beam imaging using a micro-focus source, which is usually a feature of a divergent and polychromatic X-ray source. The spatial resolution from a point source combined with the geometric magnification has routinely reached the micrometer range. Tuning the focus of the electron beam on a metallic thin film can produce an X-ray source with finer spot size down to hundreds of nanometers range. Such nano CT systems are commercially available, including the GE Nanotom and the Skyscan Ultra-High Resolution Nano-CT, with a resolution of around 200~400 nm [10]. A nano CT system based on the SEM was also developed accordingly [68]. Another type of the LB-XRT approaching 50 nm in spatial resolution was produced by Xradia (now merged into Zeiss). X-ray optics, such as condenser and Fresnel zone plates (FZP), were employed [69]. The other popular X-ray source is from synchrotron radiation due to magnetic field bending charged particles with relativistic velocity. Brilliance of the third generation SR might be ten orders of magnitude greater than that emitted from the LB X-ray tube [70]. The electromagnetic wave from SR has a wide range of spectra, covering hard X-ray to micro waves. X-ray beams used for imaging or computed tomography can be focused or parallel, polychromatic or monochromatic by tuning optics like a mirror and monochromator on the beamline. Characteristics of higher brightness and tunable wavelength bring significant improvement and possibility to X-ray imaging and computed tomography. The resolution can be below 50 nm for full field imaging or tomography by using optics like a Kirkpatrick-Baez (KB) focus mirror and FZP for the X-ray source in SR [20,71,72]. 

On the other hand, post processing of the collected XRT data is also relevant to the scanning configuration. Before the reconstruction of the collected 3D imaging dataset, wobble and shift during specimen rotation, as well as image registration, needed to be corrected first with post-processing software. Due to the differences between the LB and SR X-ray source, it has to consider a suitable reconstruction process. For LB XRT using a cone beam, a weighted back projection that considers the cone shape of the X-ray beam must be used instead of the simple back projection processing used for a parallel beam [10,73]. For this purpose, the filtered back projection (FBP) algorithm is generally used for reconstruction in the cone beam tomographs. Monochromatic X-ray beams from SR can simplify the tomographic reconstruction algorithm. The absorption of polychromatic X-ray is also an issue of concern. As low energy X-rays are easily absorbed when incoherent X-rays pass through the materials, polychromatic X-rays from the LB source gives rise to the effect of beam hardening [73,74]. This means an uneven transmission from the edge and internal regions of the sample due to the broad X-ray energy range. The penetration of higher-energy X-rays is more at the edge than the interior, so the rim looks brighter than the center of the sample, which is an artifact on the reconstructed 3D images. This artifact is generally corrected in a commercial laboratory tomography system with bundling hardware and software for optimization and correction. 

### 1.3. Timing for XRT

For LB cone beam system, one absorption contrast XRT needs to take a series of projections with a full 360 degrees of sample rotation. Hundreds or thousands of images with a specified voxel size according to the resolution requirements are recorded within a durable time, in which the sample structure is expected to be unchanged. Therefore, 3D imaging data collection by XRT is time consuming. The typical acquisition time is approximately in hours to obtain a 3D volume of 1024 × 1024 × 1024 voxels with a pixel size down to 1 μm for a LB XRT. A brighter X-ray source is more effective in resolving features with very small differences of absorptivity with a better relative signal to noise ratio (SNR). For comparison of cone beam system on the third SR, new optics and detectors used facilitated the spatial resolution reaching below 100 nm and the acquisition time in minutes to obtain a volume of 1024 × 1024 × 1024 voxels. There is no technical problem to combine XRT with an in situ experiment. However, the temporal resolution of XRT must be in the same time scale so that the structure does not change when the specified condition is applied with the measurement of property or performance testing. A higher contrast images are more easily acquired when using a brighter source. It can easily infer that the XRT by the SR source is much faster than that by the LB source in terms of the imaging speed with a proper contrast. Increase exposure time helps to improve the contrast of the imaging.

In addition to XRT with absorption contrast, which related to local density variation of the interior of materials, the chemical components of internal structure can be revealed by XRT with different approaches. Based on X-ray absorption near edge spectroscopy (XANES), a specific element or a chemical state can be distinguished by a full field imaging near its absorption edge. With the complementation of XRT, it is also feasible to show the elemental distribution not only in a 2D imaging but also in a volume of materials [75]. The 3D XANES microscopy was used to study electrochemical reduction and re-oxidation of the NiO electrode at a voxel size down to 30 nm × 30 nm × 30 nm in a FOV of 15 μm × 15 μm × 15 μm. The XANES XRT at one energy out of 13 distinct energy points spent about 1.4 h [76]. Due to the polychromatic X-ray source of the LB tube, alternative approaches for an elemental contrast tomography were developed. Dual-energy CT systems can differentiate component in materials by X-ray absorption contrast at low and high tube energies. However, extra data processing steps and calibration are required. Instead of changing tube energy, Ross-pair filters can also be utilized to produce tunable X-rays with defined energy bandwidths [77]. In terms of the K-edge energy of Rh, a combination of two filter pairs of Nb/Mo and Pd/Ag was selected for the LB XRT to identify the Rh in the Al foam. The exposure time of 15 seconds for one projection is acceptable for the LB-XRT. The other approach by employing a hyperspectral detector has much more potential applications for 3D chemical imaging. Every pixel of the newly developed detector is able to detect individual photons and extract quantitative hard X-ray spectra [78], and obtain higher spectral statistics with optimized retrieval method [79]. Such spectroscopy LB-XRT shows advantages of discrimination of the chemical components with different X-ray energies [80]. It was demonstrated in the characterization of the metallic catalyst in a porous structure and in the identification of the inclusion phases in an ore sample [81]. Another advantage of the spectroscope LB-XRT is that only one scan is needed to acquire chemical information in a 3D volume, and the scanning time is similar to normal LB-XRT.

### 1.4. Effective Factors of LB-XRT 

The brightness of the X-ray sources is not the only factor for in situ XRT. The quality of the imaged projections also relies on the penetration of the X-rays through the material by considering the absorption for a proper sample thickness. In principle, hard X-rays can transmit most materials with the energy spectrum from keV to a hundred keV. However, limitations exist for in situ experiments with the LB or SR-XRT, especially for the samples with high density such as ferrous metals and alloys, copper alloys and refractory alloys, etc. To image millimeter sized samples of such kinds of materials, the X-ray energy needs to be more than a hundred keV. In addition to the attenuation coefficient of materials at different X-ray energy, resolution and the full pixels of the detector also constrain the choice of sample size. 50 μm is estimated to be the largest dimension for a full view of the detector with 1000–2000 pixels at the resolution of 50 nm [82]. When the available sample thickness is reduced down to hundreds or tens of micrometers, however, the in situ experiment is difficult due to sample preparation and manipulation. 

To acquire magnified image with a resolution in micrometers, X-ray microscopy with cone beam projection is available for LB-XRT by using a focused point X-ray source and geometric magnification (GM). To further enlarge the image, optical magnification (OM) of visible light converted by a scintillator was feasible in the Xradia XRT systems [83]. In a SEM, cone beam X-ray can also be generated from finely focused electrons on a thin foil target. The X-ray source size can be limited by smaller interaction volume between the electrons and the thinner target, and produce the XRT with a resolution better than 100 nm [84,85]. Higher resolution in tens of nanometers are routinely available by employing X-ray objective lens as FZP to magnify full field imaging for both LB and SR-XRT. Associated with the focus of a line emission X-ray source, hard X-rays with energy at 8 keV can resolve 30 nm lines of a gold spoke pattern by using SR X-ray imaging [86] and 50 nm thick Cu tracks on a Si substrate by using LB X-ray imaging [87]. However, it takes minutes for a single projection and days for a 3D tomography with such LB systems. Table 1 shows the capability of full-field imaging and the characteristics of XRT on some typical SR beamlines and LR facilities. A comprehensive review summarized and compared these arts on X-ray nanotomography [82]. The key limitation of LB-XRT is the brightness of the available X-ray source. Recently, increment of nearly two orders of magnitude in brightness is demonstrated by using a liquid metal target in LB X-ray source. This emerging technique can load more power on the target with better heat dissipation and without melting anymore [88]. It is expected to enhance the capability of LB-XRT for faster tomography with higher resolution. 

One also needs to take the X-ray absorption differences of the material components into consideration. On one hand, large absorption differences may result in the over exposure due to high density phases. Suitable energy selection for the SR-XRT can be utilized to resolve small concentrations of the specified element in 3D volume. Dual or more energies of the SR X-ray can be used to increase sensitivity to tell the features in different phases. With the help of the sharp variation at the elemental absorption edge, the enhanced contrast provides information on the chemical difference. On the other hand, if the X-ray attenuation of different phases is similar, the absorption contrast is difficult to tell any significant differences of the phases in the grey level. The techniques of X-ray phase contrast imaging (PCI) can be applied to emphasize the appearance of the refraction at the phase interfaces. Coherent X-ray beams from SR can resolve the phase information in a straightforward approach with several mature techniques such as interferometry, propagation, edge-illumination, and grating-analyzer, etc. The propagation method of the PCI is much more easily and commonly applied to SR-XRT. Phase ring [87,93,94] and increment of propagation distance [80] are usually employed for phase contrast imaging for LB-XRT with a polychromatic cone-beam based on absorption contrast. The methods for the reconstruction of phase contrast tomography have two classes, phase retrieval and direct methods. The phase retrieval is to derive the refractive part of the X-ray through a sample and produce differential phase contrast imaging [95]. The direct method directly obtain the refractive index from the in-line plane intensity without intermediate step of phase retrieval [96]. The filtered back projection (FBP) algorithm is also commonly used for the reconstruction of such phase contrast images, by which reconstruction is processed in one step. Alternatively, an iterative algorithm can produce good reconstruction by using fewer and noisy images. A fast 3D reconstruction strategy was recently developed to perform reconstruction in 3D rather than slice-by-slice [97].

The unique characteristics of synchrotron radiation X-rays play a very important role in the advancement of in situ XRT for materials research. Environmental fixtures can be settled in the SR imaging beamline, and are employed to simulate a similar condition under service circumstances including mechanical, thermal, electrical, and electrochemical environments. Most recently, SR-XRT is able to monitor dynamic processes at high temperature. Powder sintering or liquid droplet nucleation can be recorded in 3D in real time [71]. One XRT with a voxel size about 100 nm^3^ could be completed within 20 s. The temporal and spatial resolution of high speed SR-XRT has opened new windows for in situ experiments to investigate the dynamic evolution of the structures in a nanoscale range.

Although there are many advantages of SR-XRT, access to the facilities is limited by the availabilities of scheduled beam time. LB-XRT systems will help to fulfill the growth in usage for specified applications with in situ experiments. LB-XRT is currently undergoing fast development and growth with various applications. Some companies produce commercial LB-XRT systems with continuous improvement for conventional X-ray sources and optics. Routinely, available spatial resolutions have fallen into the sub-micrometer and even nanometer range. For in situ experiments, the add-on accessories can be self-made in terms of the specified object for loading, heating, charging, and so on.

### 1.5. Studied Cases with In Situ XRT 

Most in situ experiments investigate the performance of materials, whether in a modality of a time-lapse or an interrupted in situ, can be carried out with LB-XRT and SR-XRT [98,99]. Heating and cooling apparatus were used for the materials processing studies. The in situ XRT clearly exhibited the evolution of solid/liquid mixtures and dendritic growth, and can produce real digital structures for the simulation of phase-field methods. The dendritic coarsening was mimicked in 3D view, and the speed of the interface movement could be calculated [100]. The deformation response of highly porous materials during loading was studied by in situ XRT [15,16]. The evolution of the cellular microstructure was characterized quantitatively, and the deformation mechanisms deduced by finite element simulation with the meshed model from the XRT. Damage in materials due to loading or fatigue has also been a favorable subject for in situ XRT studies [5]. The mechanisms of damage formation such as cavitation, fracture, micro-cracking, fatigue cracking, and stress corrosion cracking are proposed from a 3D viewpoint. Failure parameters of fracture and damage have been extracted and examined with the quantitative analysis of tomography images. The internal deformation of different types of materials during loading and heating were quantified and analyzed with the implementation of self-designed loading and heating equipment for the XRT [67]. SR-XRT is favorable for these in situ experiments with its advantages in brightness, monochrome, or energy options, and even space for the accessories of the in situ instrumentation. However, in situ LB-XRT experiments need to consider more limitations, e.g., the contrast from the polychromatic source, and the spaces for the sample coupled with the process and the condition control for a static and stable state during XRT acquisition.

The following review will focus on our recent works with LB-XRT exploring the mechanical, physical, chemical properties, and the correlated response of microstructures with the change of environments including mechanical loading, heat treatment, electrical treatment, corrosion, and electrochemical reactions for some typical materials. In addition, the latest advancements of in situ experiments with nano scale LB-XRT from the work of Patterson et al. will also be introduced [31].

## 2. Experimental Method 

All XRT works were carried out on our LB system, an Xradia Versa XRM-500 system (Carl Zeiss X-ray Microscopy Inc., Pleasanton, California, United States). This cone beam system was operated with the voltage in a range of 50–160 keV. The generated point X-ray source utilized Bremsstrahlung continuum spectrum. Transmitted X-ray through the sample with the geometric magnified projections traveled down to detector. The X-ray was then converted to visible light by a scintillator. The image signal magnified by optical lens was recorded by a 2000 × 2000 CCD camera (Andor Technology Ltd., Belfast, Northern Ireland, UK). The absorption contrast was imaging for the internal structure investigation in terms of the density difference from compositions, phases, or defects like voids, cracks, etc. Generally, 1600~2000 projections with a 360° rotation were taken for one 3D volume with a pixel size in the range of 0.5~40 μm. All of the datasets were performed a correction of beam hardening and then reconstructed by the bundled software kit with the FBP algorithm. Reconstructed 3D tomography data were visualized and processed with Avizo software (V7.1, Visualization Sciences Group, Bordeaux, France). through which segmentation and quantitative analysis can provide clear and solid information of the internal structure as the concern of the investigated properties. The thickness of the samples used in the following cases was carefully determined with the consideration of the attenuation of the investigated materials and checked by real XRT prior to subsequent in situ study. The properties and performances varied case by case. The experimental details are briefly described in each case. One can find more information in the referred literature of the specified cases.

## 3. LB-XRT Examples

In this section, several typical examples were selected and introduced to show what role in situ LB-XRT can play in the understanding of the correlation of materials property and performance with internal structures evolvement. The focus was the new insights obtained from in situ LB-XRT compared to the traditional characterization techniques. These examples covered some developing advanced materials such as additive manufacturing titanium alloys, zirconium-based bulk metallic glasses, third-generation single-crystal nickel-based superalloy, iron-based amorphous coatings, and new degradable biomaterials, and their key properties and performance under service environments including mechanical loading, high temperature, corrosion, electrochemical conditions, etc.

### 3.1. Mechanical Loading

Metallic cellular structures have potential applications in the human body if porous architecture, improved fatigue properties, and high energy absorption capability can be simultaneously satisfied. However, homogeneous cellular structures always own mutually opposing properties between porous architecture and mechanical strength. The topological design of the porous material may solve this problem. Zhao et al. illustrated that functionally graded Ti-6Al-4V interconnected meshes fabricated through additive manufacturing not only manifested high fatigue strength, but also integrated low density and high energy absorption, which could not be achieved by the ordinary uniform meshes [101]. Considering the coarse surface roughness of the mesh struts and requirements to see failure evolution, two kinds of in situ XRT experiments were used to elucidate the basic principles responsible for the unique mechanical behavior of the graded meshes. 

**Figure 1:**
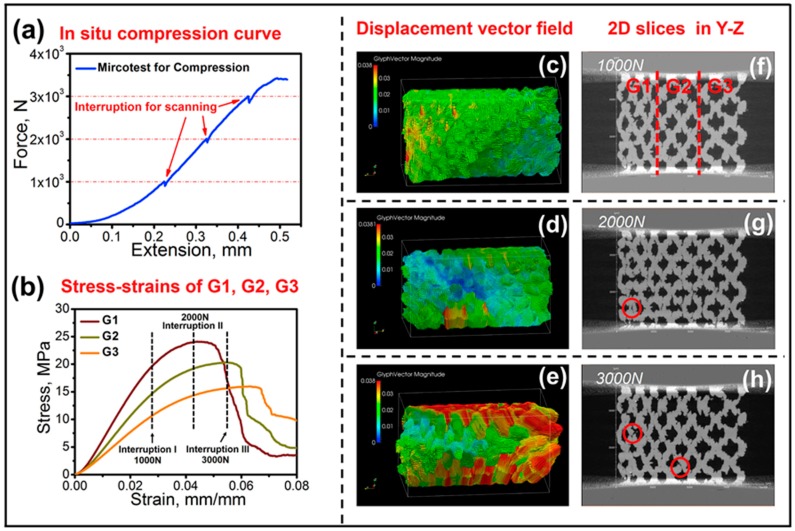
(**a**) Force-extension result acquired from in situ compression, (**b**) stress-strain behaviors of individual uniform meshes, (**f**–**h**) 2D slices showing the site of cracks marked in red circles, and (**c**–**e**) displacement vector fields of the graded structures corresponding to three interruptions during in situ compression. From the displacement maps, a phenomenon of the transition of the maximum deformation sites could be observed [101].

The first in situ experiment was a uniaxial compression test on the specimens with a dimension of 15 × 30 × 30 mm^3^ at a displacement rate of 1 × 10^−3^ mm/s [101]. The loading direction was perpendicular to the graded direction. The test was stopped at 1000 N, and the specimen was scanned using XRT. Then, the specimen was continually loaded to 2000 N. Such an interruption scanning was adopted at 1000 N, 2000 N, and 3000 N, respectively, in Figure 1a. The digital volume correlations (DVC) technique running on Davis platform offered by LaVision (LaVision GmbH, Göttingen, Germany) was used to analyze the displacement and strain field of the graded meshes based on the XRT results [101]. The color in Figure 1c–e represents the glyph vector magnitude in ParaView software. The 2D XRT slices in Figure 1f–h were used to track the sites of cracks generated at 1000 N, 2000 N, and 3000 N, respectively. The results indicated that the G1 mesh endured a process of the highest stress (Figure 1b), the highest strain (Figure 1c), cracks formed (Figure 1g), and the modulus decreased. Then, the G2 mesh successively exhibited a similar process followed by the G3 mesh. Under compressive load, such a non-uniform deformation behavior in the entire meshes originated from the graded mechanical properties of the G1, G2, and G3 meshes. The G1 part had the highest modulus and strength, so it could support the highest stress. Once cracks nucleated in the G1 part, the redistribution of the stress occurred. While many studies captured the outer or surface deformation features of graded meshes during compression test by a camera or scanning electron microscopy [102,103], the results here showed the interior deformation characteristics of graded meshes during compression by in situ XRT. This internal information gave new insights into how the substructure of graded meshes deformed and the crack nucleated in microscale while the entire sample was under elastic deformation in macroscale.

**Figure 2:**
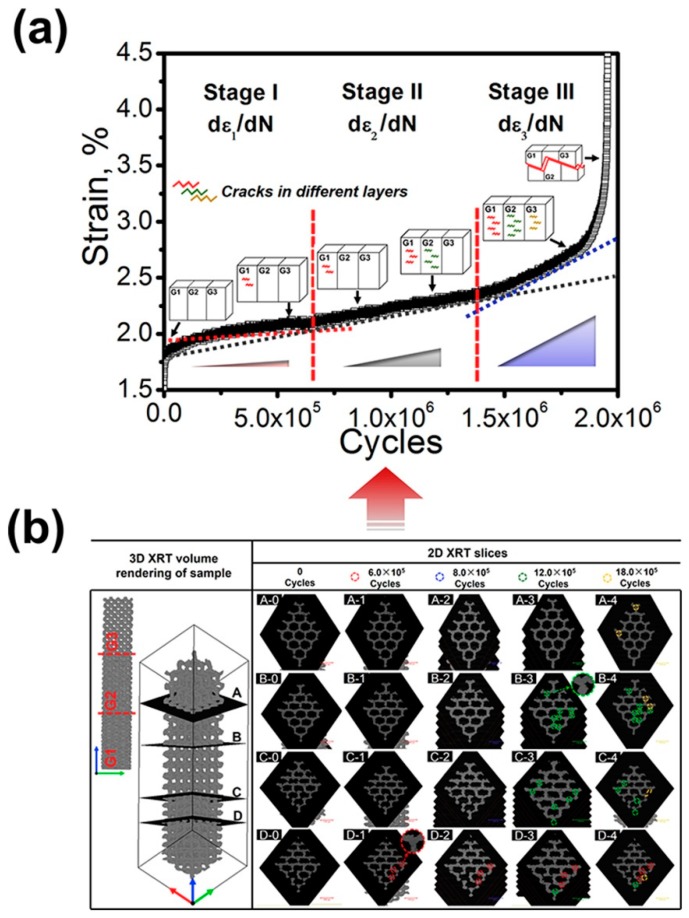
(**a**) strain-cycle curves of the graded mesh, (**b**) XRT images of the graded meshes which are corresponding to the arrow points shown in (**a**). 2D slices A, B, C, and D are located at the G3, G2, and G1 part is shown specifically sitting in the volume rendering of the sample. The numbers 0, 1, 2, 3, and 4 are in accordance with the 5 arrow point, respectively. The colored dash cycles in (**b**) indicate the sites of the cracks observed at each arrow point [101].

The second in situ experiment was a high cycle compressive fatigue test on the specimens with a dimension of 2 × 6 × 10 mm at a stress ratio, R, of 0.1 and a frequency of 10 Hz [101]. The loading direction was also perpendicular to the graded direction. The strain-cycle curves (ε-N) of the graded cellular structures in Figure 2 could be divided into three stages according to the slope of the curves in Figure 2a. In situ XRT was employed to find the sites of the cracks during cyclic compressive fatigue deformation in Figure 2b. In stage I, the cracks first formed in the G1 part. In stage II, the old cracks in the G1 part propagated at a slow speed while new cracks were detected in both the G1 and G2 parts. In stage III, additional new cracks were detected in the G3 mesh when the fatigue cycles achieved the sudden increase strain zone. The behavior of nucleation and propagation showed that the cracks were prone to initiate in the G1, G2, and G3 parts than near the transition zone, even though the distribution of the stress at the transition zone was not continuous. The progressive initiation of the cracks in the order of G1, G2, and G3 made a big difference from the crack behavior of the uniform meshes where the cracks propagated quickly until the whole mesh sample fractured once the cracks initiated. It was implied that the graded meshes with continuous stress redistribution could retard the abrupt collapse during fatigue, which might also be the origin of the unique cyclic ratcheting rate for the graded meshes in Figure 2. It should be noted that the theoretical analyses also showed that the fatigue behavior of the graded meshes was mainly determined by the stress distribution in the constituent meshes during cyclic compressive fatigue deformation [101]. Such a stress distribution phenomenon for graded meshes was the origin of the combination of low density, high fatigue strength, and high energy absorption, which could not be simultaneously achieved according to the reported metallic cellular structures with uniform density [104,105,106].

The above two experiments displayed the internal deformation and fracture behavior of graded meshes under different loading mode. One was loaded at a constant displacement rate; the other was loaded and unloaded under compression cyclic mode with a stress smaller than 3.6 MPa. These in situ XRT results would enrich and deepen the understanding of the relationship between topological designs of graded structure and different properties.

**Figure 3:**
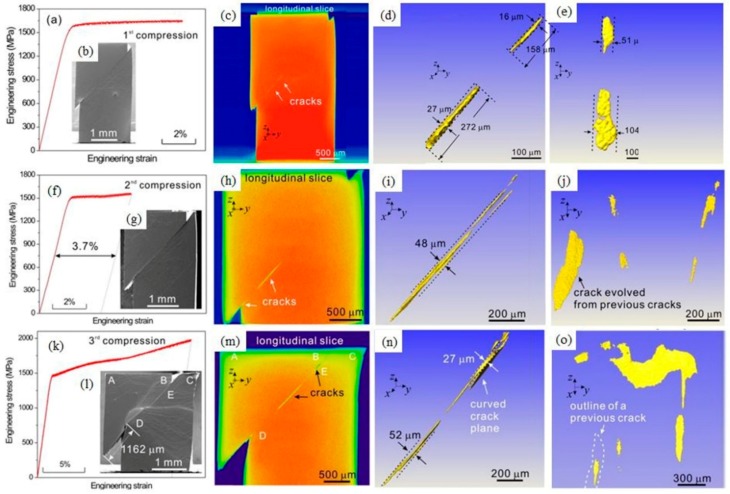
Shear band cracking behavior in an in situ compression test. The compression, SEM, and XRT results in the rows after the first stop (**a**–**e**), after the second stop (**f**–**j**), and after the third stop (**k**–**o**), respectively [107].

For metallic glass (MG), how the shear band (SB) cracks if there is no negative pressure is still an open question. To answer this question, three uniaxial compression tests at a strain rate of 10^−4^ s^−1^ using the same sample with dimensions of 2 × 2 × 4 mm for the ductile Zr_65_Fe_5_Al_10_Cu_20_ (at %) MG was conducted [107]. After the first compression, the specimen was taken away to be scanned using XRT. Then, the specimen performed the second compression, followed by the second XRT scanning and the third compression and XRT scanning. XRT was used to reveal the evolution behavior of the interior SB cracks during in situ compression in Figure 3. To our knowledge, this XRT work was the pioneering study of 3D imaging on internal shear-banding cracks and their evolution. Several key findings were obtained from the longitudinal 2D slices and 3D extracted cracks. A phenomenon of discontinuous nucleation, linkage, and propagation of the crack, as well as shrinkage or closure was seen along the major SB. The long-narrow-thin 3D crack with a thickness of 16–27 μm can be regarded as the affected zone of shear-banding, which was much larger than the initial SB (~10 nm). Some unique features such as non-coplanar behavior and the largest crack with a curved plane were also captured. These in situ XRT results accompanied by SEM results verified that SB cracking may be traced back to one or a combination of the three sources including the excess free volume [108,109,110], shearing of non-planar SB [111,112], and SB interaction. The reported experiments in the literatures usually obtained limited SB cracking results from either fracture surface morphology or polished sections [111,113,114]. This in situ XRT results contributed new findings on how SB evolves into crack under compression loading mode.

**Figure 4:**
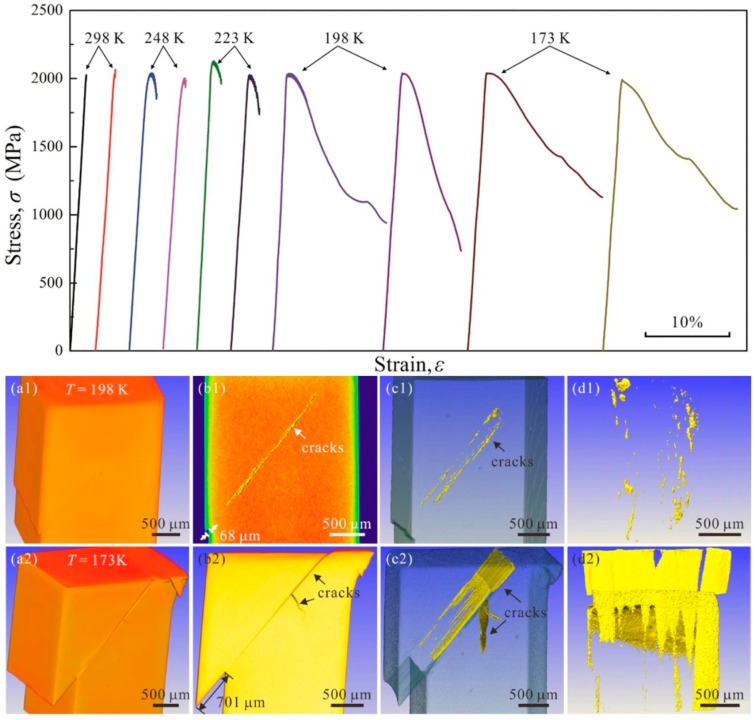
Typical engineering stress–strain curves under compression at temperatures from 298 K to 173 K showing the influence of testing temperature on the stress-strain behavior of Ti32.8Zr30.2Ni5.3Cu9Be22.7 metallic glass and the XRT results corresponding to the samples at 198 K (**a1**–**d1**) and 173 K (**a2**–**d2**). (**a1**–**a2**) the global 3D volume renderings of the samples. (**b1**–**b2**) 2D slice or cross-section showing the internal cracks well located in the shear band plane. (**c1**–**c2**) and (**d1**–**d2**) 3D extracted internal cracks observed from different perspectives. The XRT volume renderings together with the 3D internal cracks show the temperature effect on the deformation of the MG sample [115].

Generally, most materials will have a more brittle behavior at low temperatures when compared with a high temperature. Surprisingly, when a relatively brittle Ti_32.8_Zr_30.2_Ni_5.3_Cu_9_Be_22.7_ (at %) MG was tested under compression at a strain rate of 10^−4^ s^−1^ using the sample with dimensions of 2 × 2 × 4 mm, both the plasticity and the yield strength were improved while the apparent softening rate decreased with the decrease of the temperature from room temperature to 173 K as shown in Figure 4 [115]. The XRT was used to extract the 3D internal cracks to understand the shear band cracking behavior of the MG. Two samples deformed at 198 K and 173 K unloaded at different periods of compressive deformation showed interesting XRT images in Figure 4. Scattered and small cracks were found in the shear band plane for the sample with a 68 μm shear offset tested at 198 K, while long and large cracks were observed for the sample with a 701 μm shear offset tested at 173 K. This illustrated that the testing temperature accompanied by the amount of shear plastic deformation influenced the crack evolution. In addition, a split crack was detected in the sample at 173 K in Figure 4c2–d2, which may have originated from the local bending moment. The 2D slices and 3D XRT images also showed many uncracked areas which carried the load during the test. These uncracked areas would decrease, while total stress reduced when the plastic deformation increased accordingly. As a consequence, the apparent softening behavior occurred. Within several samples instead of one of the same samples, these XRT results clearly demonstrated that the apparent softening was more likely to come from SB cracking rather than the commonly accepted view of SB dilation.

### 3.2. Heat Treatment

The high-temperature capabilities make single-crystal (SX) nickel-based superalloys one of the material choices in turbine blades. Key properties such as the fatigue and creep properties of SX superalloys are critically affected by micro-pores. Micro-pores are associated with solidification and heat treatment. In the final stage of solidification, the liquid metal contracts, and gas solubility sharply decreases. The pores formed in this process are called S-pores. The solution heat treatment time and temperature can also generate micro-pores named H-pores. Although many studies have reported the growth of micro-pores at high temperatures [116,117], the S-pores and H-pores in these studies were treated as the same micro-pores. How the S-pore and H-pore evolve and their respective fundamental mechanisms are still unsolved issues [118]. 

**Figure 5:**
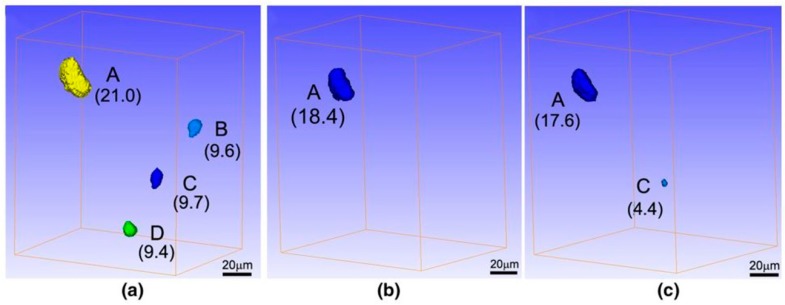

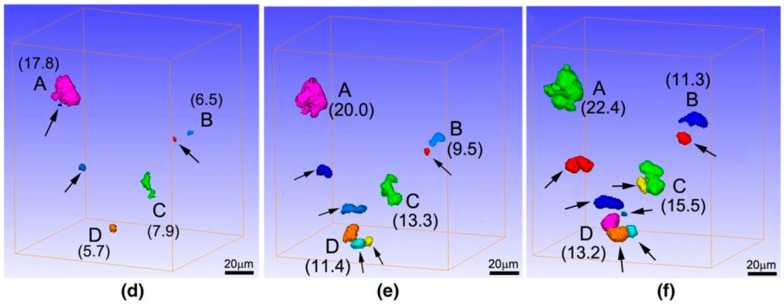
XRT volume renderings of micro-pores in a same sample with a heat treatment at 1603 K for (**a**) 0 h, (**b**) 1 h, (**c**) 4 h, (**d**) 7 h, (**e**) 12 h, and (**f**) 20 h. The equivalent diameter (μm) of the S-pores is also given [118].

In situ XRT was performed on the same sample with dimensions of 10 mm in length and 1 mm × 1 mm in cross-section to characterize the S-pores and H-pores and quantitatively study the evolution behavior of each pore during solution heat treatment at 1603 K for 1, 4, 7, 12, and 20 h, respectively. The samples were first scanned using XRT, then sealed in vacuum tubes and exposed at 1603 K for 1 h for the solution heat treatment. After that, the samples were taken out to be re-scanned using XRT. This process was repeated several times with the only difference of the solution heat treatment was time, by substituting 4, 7, 12, and 20 h for one hour [118]. The XRT volume renderings of the micro-pores in the same sample showed the characteristics during the solution heat treatment in Figure 5. It is quite interesting to observe that new H-pores (indicated by the arrows) formed and grew during the solution heat treatment. For the S-pores, the volume fraction gradually decreased at 1603 K at 1 and 4 h, then increased at 7, 12, and 20 h. For the H-pores, the volume fraction increased during the entire solution process. Considering that the cross-diffusion of elements was imbalanced, a vacancy would form and diffuse during heat treatment. Such vacancy behavior was believed to be related to the evolution of both S-pores and H-pores. The micro-pores may have a maximum volume fraction during the solution heat treatment according to the experimental results [116] and theoretical study [119]. Such a maximum was not observed in the present solution heat treatment at 1603 K for less than 20 h. If the solution heat treatment time was further increased, the maximum would eventually occur. 

### 3.3. Electropulsing Treatment

**Figure 6:**
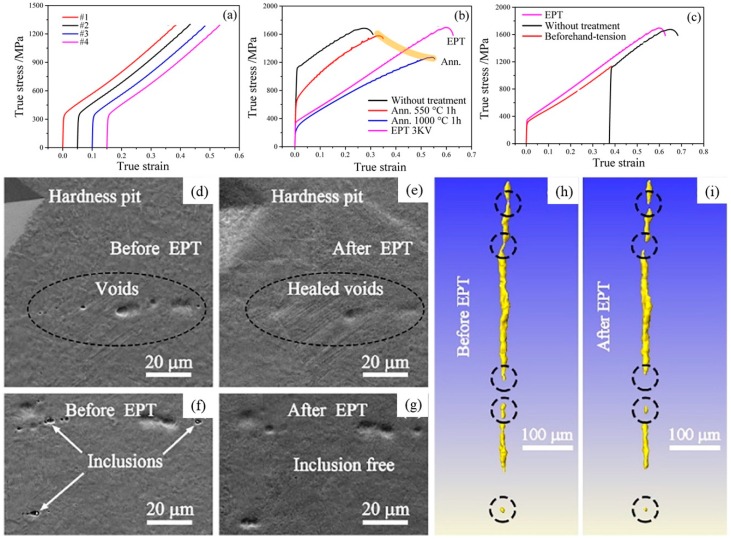
(**a**–**c**) tensile results, (**d**–**g**) SEM images, and (**h**–**i**) XRT volume renderings of a crack for Fe–22Mn–0.9C TWIP steel specimens. These results show the healing effect with EPT, which is superior to the annealing technique [120].

Twinning-induced plasticity (TWIP) steel, as a future candidate for the aerospace industry, greatly combines high tensile strength and uniform elongation by means of the high strain-hardening capacity. If TWIP steels are subjected to plastic deformation, the initiation of micro-voids could undermine the strain-hardening capability and result in premature failure. Improving the mechanical properties is expected by eliminating the micro voids using electropulsing treatment (EPT) [120]. Figure 6a–c shows an interesting phenomenon where EPT could recover the strength and plasticity, but annealing treatment had no such ability. The evidence on the sample surface as shown in Figure 6d–g proved that the voids could indeed be healing as well as that the inclusions could be eliminated by EPT. This led to the increase of the strain-hardening capability and resulted in the recovery of strength and plasticity of the TWIP steels samples when compared with those annealed counterparts. One may doubt that the void healing effect may be a pseudomorph on the sample surface due to the quasi in situ processing.

To clarify the internal structure change, in situ XRT was used to verify the healing effect of EPT. A specimen with dimensions of 1.8 × 1.8 × 50 mm was scanned using XRT before EPT. Then, the specimen was electropulse treated using self-made equipment with a capacitor bank discharge circuit with the discharge voltage of 250 V and current pulse duration of 400 ns at room temperature. After EPT, the specimen was re-scanned using XRT to detect the evolution of the macro voids and cracks.

Figure 6h,i show the XRT images of a long 3D crack composed of several short cracks in a strained TWIP steel specimen before and after EPT, respectively. The overall length of the crack decreased from about 400 μm before EPT to 300 μm after EPT. In addition, a macro-void at the bottom in Figure 6h,i became much smaller after EPT when compared with that before EPT. Therefore, the XRT results proved that macro voids in the interior of the material could indeed be healed by EPT in the TWIP steel. The healing effect of EPT may originate from two aspects: one is the elevated temperature near the crack, and the other is the thermal compressive stress around the crack [121]. The material near the crack may be molten or expand, while that far away from the crack remains solid or did not expand so much [122]. Thus, the melted material may endure compression and be gradually compressed into the crack [123]. Thus, the cracks could be healed after cooling. This healing process may also be used to explain the healing of the macro voids by EPT. This damage-healing method could be expanded to heal the damage in those engineering alloys under cyclic deformation and prolong their service life.

### 3.4. Protective Coating from Corrosion

Thermal sprayed coatings have been widely used in industry to protect the surfaces of metals and alloys against corrosion and wear. The corrosion resistance of the coatings is related to the coating porosity, which can be categorized as through-porosity and non-through porosity on the basis of its behavior in the corrosion of the coated materials. According to the potentiodynamic polarization curves of the Fe_49.7_Cr_18_Mn_1.9_Mo_7.4_W_1.6_B_15.2_C_3.8_Si_2.4_ amorphous coating with different thicknesses, the anodic current density increased when the coating thickness decreased from 700 μm to 60 μm when the potential was higher than 0.3 V_SCE_ [124]. This phenomenon was inferred to be related with the through-porosity, which could lead to direct paths between the substrate and corrosive environment. The electrolyte easily penetrated the substrate from through-porosity, and caused the substrate to dissolve once the potential was above the pitting potential of the substrate. However, direct evidence was needed to confirm the substrate corrosion beneath the coating.

In situ XRT technique was used to observe the substrate corrosion evolution beneath the coating before and after electrochemical measurements. The initial specimen with a coating thickness of 150 μm and coating surface of 1 mm × 1 mm was scanned using XRT, and then taken out to be dynamically polarized in the anodic direction at a rate of 0.333 mV/s and interrupted at 0.7 V_SCE_ for the following XRT measurement. To clearly demonstrate substrate corrosion, a further potentiostatic polarization test was performed on the specimen at 0.7 V_SCE_ for 10,000 s, and then scanned by XRT. All XRT scanning was configured with the same parameters. 

**Figure 7:**
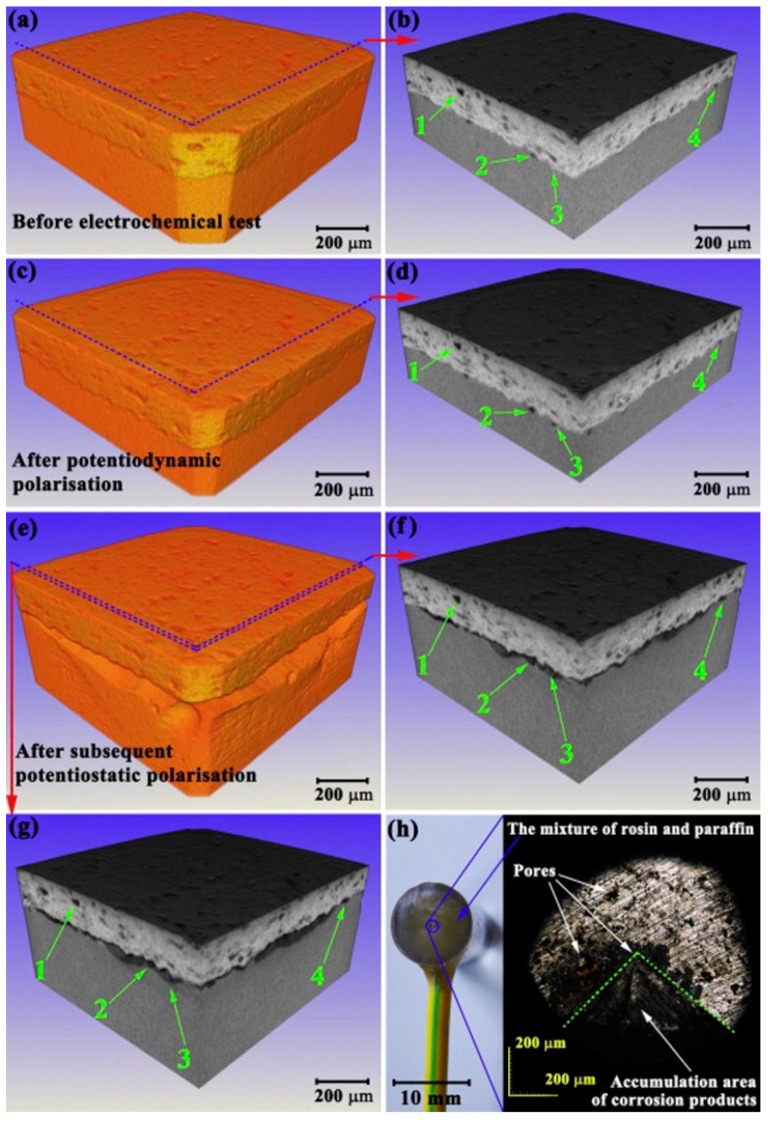
(**a**–**g**) 3D XRT images showing the substrate corrosion evolution beneath the coating during three stages, and (**h**) coating surface morphology showing the accumulation of corrosion products [124].

The 3D XRT volume renderings are given in Figure 7a–g for the same coated sample acquired before and after the potentiodynamic polarization test and subsequent potentiostatic polarization. A preferential site in the substrate for corrosion at the interface between the coating and substrate was found and tracked. A large corrosion pit was verified at the last stage. Focusing the view on the coating surface, we could see that the corrosion products accumulated and piled, which may be attributed to the continuous flow of corrosion products from the through-porosity (Figure 7h). This evidence helps confirm that the abrupt variation of the anodic current of a thinner coating at the potentials above 0.3 V_SCE_ originated from the through-porosity. Compared with non-through porosity, through-porosity could cause increased detriment to the coated material [125,126]. Until this work, direct evidence of substrate corrosion beneath the coating was captured. If the presence and the role of through-porosity is known, the critical coating thickness can be estimated, which is beneficial for the development of advanced metallic coatings for corrosion resistance.

Magnesium-based biodegradable implants can be used in the fields of orthopedics because of their safe degradation behavior and no need to remove after bone healing. The application limitation mainly focuses on the initial rapid degradation, which will lead to hydrogen bubbling and loss of mechanical support, and then, decrease the bone growth. Surface modification may solve this potential issue for future applications. Han et al. [89] fabricated an Mg–1.5  wt % Sr alloy with Sr–Ca–P containing a micro-arc oxidation (MAO) coating to evaluate the role of the coating on the degradation behavior of the implants.

Since in vitro degradation measurements including mass loss, hydrogen evolution, and electrochemical behavior cannot draw complete maps for the in vivo status, 3D XRT with a pixel size of 25.94 μm was applied to visualize the in vivo degradation amount of the distal femora of rabbit at the implantation periods of eight weeks post-surgery. The dimensions of the rods for the MAO-coated Mg–Sr alloy and the control Mg–Sr alloy without coating were Φ 1.5 mm × 20 mm.

**Figure 8:**
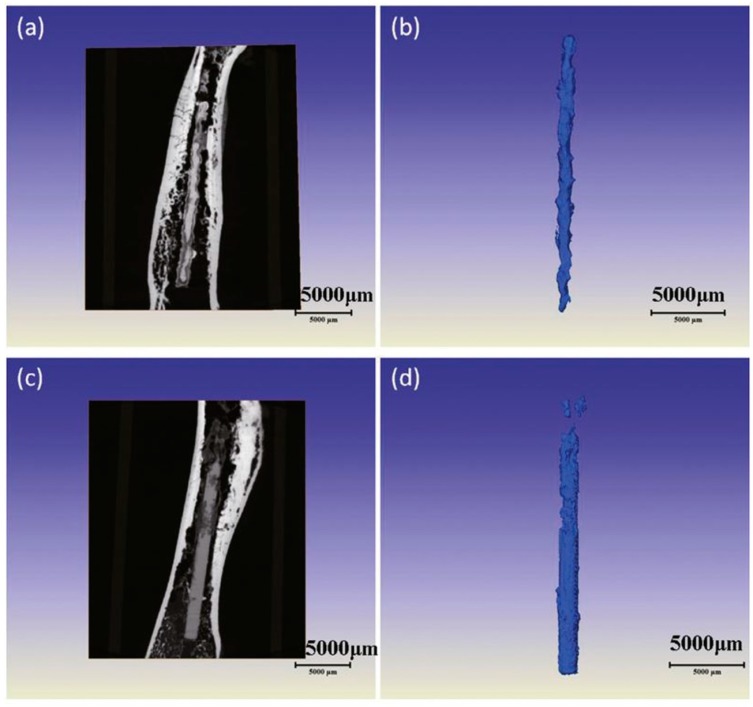
2D slices and 3D volume renderings of (**a**,**b**) Mg–Sr alloy and (**c**,**d**) Mg–Sr alloy with Sr–CaP coating visualized in vivo using the XRT technique after implantation of 8 weeks in rabbit femur [129].

Figure 8 displays the 2D slices and volume renderings of the remaining Mg–Sr alloy with and without Sr–Ca-P coating implants as well as the surrounding bone tissue in the rabbit distal femur. For the Mg–Sr alloy, pitting corrosion occurred around the entire rod after eight weeks, and only the central area remained in the original state. In comparison, uniform corrosion was observed on the Mg–Sr alloy with MAO coating except for some pitting areas. The lower part of the rod still maintained its integrity while the upper part in the proximal site showed severe degradation. Similar phenomena were also reported by Gu et al. [127]. After the calculations according to the uncorroded parts using the quantitative XRT results, the Mg–Sr alloy with MAO coating showed a degradation speed of 0.75 mm/year while the speed of the Mg–Sr alloy without coating was about 1.3 mm/year. These results manifested that the MAO coating can play a role as a corrosion barrier [128] and help to keep the integrity of the Mg–Sr alloy in vivo [129].

### 3.5. Electrochemical Reaction

The Li-S battery is regarded as an excellent potential candidate for high capacity energy storage devices [130,131]. The limitations of advanced high energy density Li-S batteries in real use mainly focus on two factors: low sulfur loading and low sulfur content when the electrode is only considered. This problem impedes the commercial application of Li-S batteries. In the common sense of the community of Li-S batteries, it is hard to simultaneously increase the electrochemical performance and sulfur loading. Therefore, it is still an unresolved key issue on how to increase the sulfur loading and sulfur content without the sacrifice of the electrochemical performance. Design cathodes with a 3D structure may be a solution to this problem [132,133,134].

3D graphene foam electrodes were subtly designed to achieve high sulfur loading for high energy density Li-S batteries. The highest sulfur loading was about 10.1 mg cm^−2^. During the XRT tests, two samples were used. One sample of 3D graphene foam electrodes before cycling was cut into pieces and scanned using XRT. The other sample was assembled into 2025-type stainless steel coin cells. After 1000 cycles, the graphene foam electrodes were taken out to perform XRT scanning at different length scales. 

**Figure 9:**
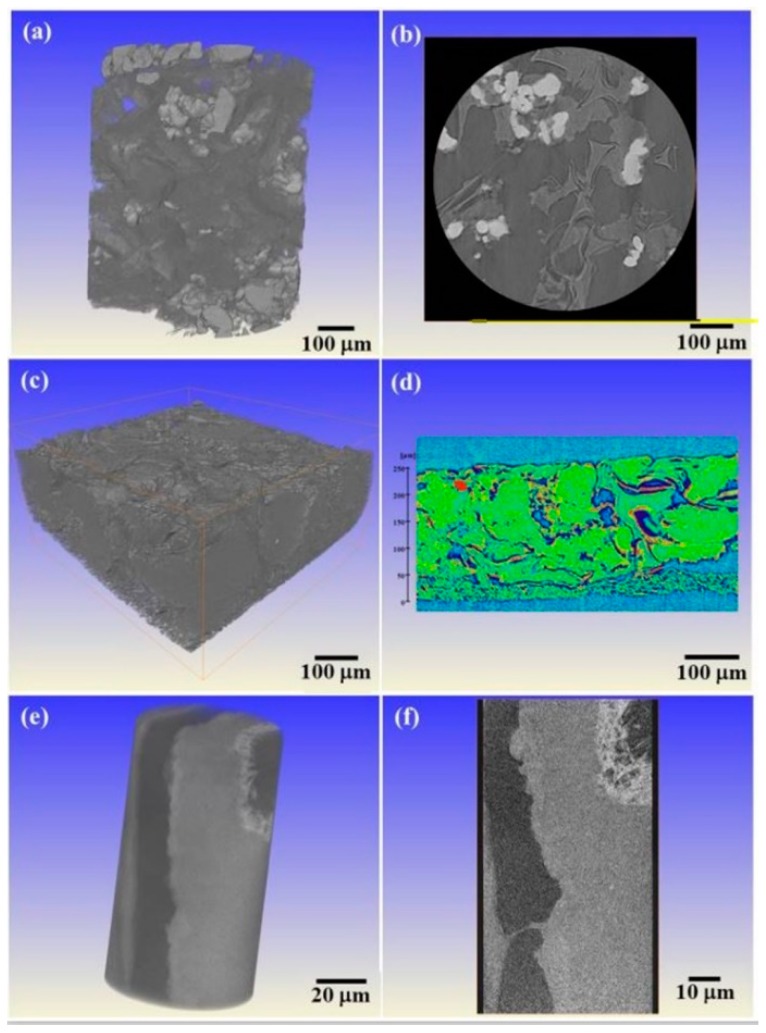
3D XRT volume renderings and 2D slices of the 3D grapheme foam electrodes with 10.1 mg cm^–2^ sulfur loading: (**a**,**b**) original state; (**c**–**f**) after 1000 charge/discharge cycles; (**a**–**d**) the pixel size is about 0.7 μm; and (**e**,**f**) the pixel size is about 64 nm.

Before cycling, we could visualize the 3D structure of graphene foam using the XRT technique with a pixel size of 0.68 μm in Figure 9a,b. It was quite easy to distinguish the sulfur particles from the carbon black and graphene. The sulfur particles were located at the network of the 3D graphene foam. The areal capacity of the electrode with the highest sulfur loading could achieve 13.4 mA h cm^−2^. Such an excellent value of the electrochemical performance was superior to the published results for the Li-S electrodes. After 1000 cycles, there was still a large amount of sulfur in the active material layer in the electrode. Both the shape and size of the sulfur greatly changed in Figure 9c,d. The pixel size of 0.68 μm was not enough to provide clear details of the sulfur, so an Xradia 800 ultra was used for ultra-high spatial resolution. Figure 9e,f present the XRT results with a pixel size of 64 nm. It can be clearly seen that the size of the large sulfur with a needle-like shape was in a range of 150 nm to 20 μm while the size of small sulfur is less than 150 nm. All the sulfur distributed on the outer surface of the slurry. This work displayed the sulfur morphology and distribution change before cycling and after 1000 cycles. Such a 3D XRT characterization on the electrodes of Li-S batteries after 1000 cycles has never been done before. Researchers have been more prone to capture the changes during different stages of one cycle using the 2D synchrotron XRT method [135] or have investigated the sulfur degradation behavior during the initial 10 cycles using X-ray phase contrast tomography [136]. Nevertheless, the above results proved that the design of a 3D graphene foam based flexible electrode can satisfy the demand for the Li-S battery with high energy density, high power density, and long cyclic life to some extent.

### 3.6. Nano XRT In Situ Experiment 

Most works of in situ LB-XRT under mechanical loading, heating, or harsh environment have a limited resolution at the micrometer scale. Recently, the spatial resolution of the in situ LB-XRT has been greatly improved. It was claimed that the initiation and propagation of the crack could be accurately probed at the scale of 200 nm [31]. This progress was achieved by using an in situ mechanical stage where a load arm was driven by a piezo motor. This stage had been integrated into the nano X-ray tomography systems. Three operating modes including nanoindentation, compression, and tension were supported on this assembly. Data could be recorded in a quasistatic, interrupted in situ manner while one 3D tomographic data acquisition needed several hours. 

**Figure 10:**
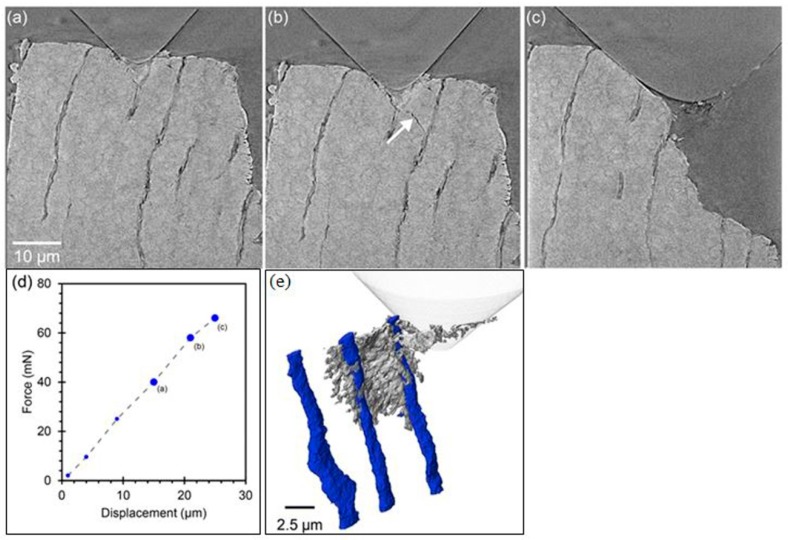
(**a**–**c**) 2D slices showing crack evolution corresponding to the different stages of indenter loading. (**d**) The force-displacement curve during the nano-indentation test. (**e**) 3D volume rendering of an extracted crack at the loading point (**b**) on the force-displacement curve showing the relationship of the crack with selected dentin tubules [31].

The crack growth in the dentin of elephant tusk under nanoindentation was investigated. It provided insights into anisotropic fracture behavior and crack-shielding mechanisms. In this in situ experiment, a cone indenter with a tip radius of 1 μm and tip angle of 90° was used to initiate and propagate cracks on a cone shape dentin sample with the height of 1 mm and top surface diameter of 50 μm. The load was applied incrementally and held in a manner of displacement control at different stages (Figure 10a–c). Zernike phase contrast mode was used with 721 projections. Each projection was exposed for 60 s with 64 nm pixel size. Figure 10a–c present continuous crack propagation as the indentation was increased. Further information was obtained from the 3D segmented image (Figure 10e). The extension of the crack was mainly in a radial direction from the indenter, which seemed to be a crack path with low energy. A deflecting crack path could also be deduced from the cracks growing in other directions. Therefore, cracking and bridging seem to be the main fracture toughening mechanisms of tubules for the tusk. This example demonstrated that enhanced insight on material performance was no longer limited in synchrotron-based X-ray tomography, and could be explored through lab-based X-ray nano-tomography.

## 4. Conclusions and Perspective

With high brilliance and monochromatic X-rays, the advantages of the SR-XRT boosted the in situ experiments for the investigation of the temporal evolution of the 3D structure coupled with the measurements of the properties. The researchers now are devoted to develop 3D tomography system with the temporal resolution stepped into seconds, the voxel size down to a few tens of nanometers, or even the combination. Meanwhile, the brightness of the X-ray tube in the laboratory constrains the advancement towards real-time in situ XRT. However, LB-XRT is also viable and easily accessible for specified research interests covering a controllable condition or environment where the structure studied can be stable and imaged by 3D tomography statically. As in the cases we showed in this review, LB-XRT is feasible for some common properties or performances of materials to correlate with their 3D structures. It can focus on, but is not limited to, research fields such as crack or damage growth during loading or electric stressing, structure and void evolution with thermal processing, composition change or transport in controllable chemical or electrochemical environment, etc.

Recently, the diffraction contrast tomography (DCT) solution was developed for laboratory-based Xradia Versa 520 system. Crystallographic information of the individual grains from polycrystalline samples can be identified and demonstrated in 3D space with colorful marks for different orientations [137,138,139]. The DCT developed in the past decade have been based on synchrotron monochromatic X-rays. It is now available in laboratories as a routine tool for non-destructive 3D grain mapping. As a complementary to the XRT in-house from absorption or phase contrast tomography, it is possible to carry out characterization and also study of the mechanisms of damage, deformation, and growth related to grains, rather than the sole phase or morphology structures.

Expanded application fields for LB-XRT need to be explored by a coordination of novel technologies. One of them nowadays attracts fast growing interest. The 3D digital volume from a real structure can be meshed and simulated by the Finite Elements tool kit to see the local strain distribution with the corresponding structure variation in the applied field. Another technique is the so-called digital volume correlation (DVC), which can be performed to measure the local displacement inside the bulk and reveal the strain field in a 3D global manner [35,140,141,142,143]. The combination of these novel techniques will play a critical role in investigating materials with heterogeneous structures such as various composites, architecture materials made by additive manufacturing, biomaterials with hierarchical structures, etc. To understand the mechanism of the initiation and extension of cracks or damage in such materials and improve the resistivity with structure tuning might be fascinating research for the LB-XCT to be used.

According to the present modality of LB-XRT, the limited brightness of the X-ray source is an obstacle that is difficult to cross for the real time 3D characterization of structure for dynamic processes in a few minutes or seconds. Instead of the hardware improvement, the optimization of data redundancy in the tomography by software development can be another route for fast tomography. This new strategy takes only effective information or compresses information with little loss. In practical operation, a new approach named the projection-based digital volume correlation (P-DVC) was proposed to make accurate reconstructions with fewer projections [144,145]. A fast tomography of a tensile test on a cast iron sample with radiography and LB-XRT realized 127 loading steps in a total time of 10–15 min, a time saving of at least two orders of magnitude. This emerging method shows a very bright future for fast tomography in-house with time resolution in seconds. 

## Figures and Tables

**Table 1:** Typical SR beamlines and LB facilities for X-ray nano tomography with full field view.

Source	Facility	Beamline/Model	Energy keV	Flux phs/s	Res. nm	Ref.	XRT
SR	ESRF	ID11	18–140	~ 10^14^	100	[89]	Monochromatic/Pink Tunable energy Minutes Tomo. Time-lapse Tomo. Absorption contrast Phase contrast
	APS	34 ID-C	5–15	5 × 10^9^	100	[90]	
	Spring-8	BL29XUL	4.4–37.8	6 × 10^13^	50	[91]	
	SSRF	B13W1	8–72.5	3 × 10^10^	100	[92]	
LB *	MXIF	Gatan XuM	9.7		200	Web.	Polychromatic/Monochromatic Hours Tomo. Interrupted Tomo. Absorption contrast Phase contrast
	IMR	Xradia Versa 500	30–160	5 × 10^8^	700	Spec.	
	IMR	Xradia 810 Ultra	8		50	Spec.	

* Data from the website (Web.) and the specifications (Spec.) measured with the facility.
